# Intercommunication between Voltage-Gated Calcium Channels and Estrogen Receptor/Estrogen Signaling: Insights into Physiological and Pathological Conditions

**DOI:** 10.3390/cells11233850

**Published:** 2022-11-30

**Authors:** Yashashwini Dinesh Subbamanda, Anamika Bhargava

**Affiliations:** Department of Biotechnology, Indian Institute of Technology Hyderabad (IITH), Kandi, Sangareddy 502284, Telangana, India

**Keywords:** voltage-gated calcium channels, T-type calcium channels, L-type calcium channels, calcium influx, estrogen receptor signaling, estrogen

## Abstract

Voltage-gated calcium channels (VGCCs) and estrogen receptors are important cellular proteins that have been shown to interact with each other across varied cells and tissues. Estrogen hormone, the ligand for estrogen receptors, can also exert its effects independent of estrogen receptors that collectively constitute non-genomic mechanisms. Here, we provide insights into the VGCC regulation by estrogen and the possible mechanisms involved therein across several cell types. Notably, most of the interaction is described in neuronal and cardiovascular tissues given the importance of VGCCs in these electrically excitable tissues. We describe the modulation of various VGCCs by estrogen known so far in physiological conditions and pathological conditions. We observed that in most in vitro studies higher concentrations of estrogen were used while a handful of in vivo studies used meager concentrations resulting in inhibition or upregulation of VGCCs, respectively. There is a need for more relevant physiological assays to study the regulation of VGCCs by estrogen. Additionally, other interacting receptors and partners need to be identified that may be involved in exerting estrogen receptor-independent effects of estrogen.

## 1. Introduction

Emerging evidence points toward the role of voltage-gated ion channels in diseases pertaining to non-excitable cells such as diabetes and cancers. Among voltage-gated ion channels, voltage-gated calcium channels (VGCCs) have gained particular attention due to the involvement of calcium in pathological processes. Since estrogen plays an important role in the pathology of hormone-related cancers and estrogen is shown to modulate L-type VGCCs, it is important to understand the interactions between estrogen and VGCCs in physiology and pathology. In this article, we provide a detailed account of VGCC regulation by estrogen and mechanisms therein wherever investigated. We also provide some new insights based on the data reviewed.

### 1.1. Voltage-Gated Calcium Channels

Electrophysiological techniques such as patch-clamp electrophysiology have led to a greater understanding of the functional properties of VGCCs such as their gating, selectivity, and permeability [1]. Based on the degree of depolarization required for the channel opening, VGCCs are classified as high voltage-activated (HVA) or low voltage-activated (LVA) calcium channels. The HVA calcium channel group consists of Ca_V_1 subfamily (long-lasting/L-type) and Ca_V_2 subfamily (P/Q, N and R type), whereas, Ca_V_3 subfamily forms the LVA T-type/transient calcium channel [2,3,4]. Since the first discovery of calcium channels by Paul Fatt and Bernard Katz in the crustacean muscle, a plethora of research has been carried out on the role of VGCCs in different cellular functions. The classification, pharmacology, and physiological functions of VGCC subtypes are well-documented [5,6,7]. The distribution of HVA L-type calcium channels (LTCCs) and LVA T-type calcium channels (TTCCs) is overlapping and is observed in a wide variety of tissues/cell types. The LTCC subfamily comprises Ca_V_1.1, Ca_V_1.2, Ca_V_1.3, and Ca_V_1.4. While Ca_V_1.1 is exclusively expressed in skeletal muscles, Ca_V_1.2 is mainly expressed in the neuronal dendrites. Ca_V_1.3 has a wide distribution which includes the heart, brain, kidney, pancreas, etc., while Ca_V_1.4 is predominantly found in the retinal cells [8,9,10,11]. Both Ca_V_1.2 and Ca_V_1.3 are involved in spontaneous firing and pacemaker activities. TTCC family comprises three channel isoforms Ca_V_3.1/α1G, Ca_V_3.2/α1H, and Ca_V_3.3/α1I. As mentioned above, like LTCCs, TTCCs also have a widespread expression including heart, nervous tissue, kidney, sperm, endocrine tissues, etc. [12,13,14,15]. HVA N, P, Q, and R-type calcium channels are expressed prominently in the neurons. The P/Q (Ca_V_2.1) type channels are highly expressed in the Purkinje cells, granule cells of the cerebellum, and mammalian brain cortex [16,17]. N-type calcium channel (Ca_V_2.2) is present in the central and peripheral nervous systems. R-type channel isoform (α1Ee) is expressed in the islet of Langerhans and kidney [18]. For detailed localization of calcium channel subtypes please see Table 1.

Since their discovery, calcium channels have gained interest due to their involvement in several pathological conditions. Here, we are describing their physiological and pathological roles briefly, for detailed reviews please see [7,31,32,33]. HVA P/Q-type VGCCs initiate vesicle release at the presynaptic terminals for neuronal communication [34]. P/Q and N-type VGCCs play a role in stimulus–secretion coupling [35]. N-type VGCCs are involved in nociception, depolarization evoked norepinephrine release, etc. [36,37,38]. R-type VGCCs are involved in the secretion of peptide hormones, long-term potentiation, exocytosis, and neurotransmitter release [39,40]. HVA LTCCs play a vital role in physiological processes such as excitation–transcription coupling, excitation–contraction coupling, excitation–secretion coupling, cardiac pacemaker activity, and visual function [6,10,11,41,42]. TTCCs play a vital role in the cardiac [43,44] and neuronal tissues [45,46,47,48]. Their roles have also been identified in hormone secretion, gene expression, cell development and proliferation, and cell cycle regulation.

P/Q-type channel activity is potentiated in diseases such as familial hemiplegic migraine and Alzheimer’s disease whereas, loss of P/Q-type channel function is observed in pathological conditions such as ataxia and absence epilepsy [49,50,51]. Dysregulation of N-type calcium channel expression and activity leads to cognitive decline as observed in the fragile X syndrome [52]. R-type (Ca_V_2.3/α1E) calcium channels are involved in both convulsive and non-convulsive seizures [53]. Recently, Schneider et al. have described human mutations associated with R-type calcium channels that contribute to early death [54]. Various L-type channelopathies have been discovered which include autism, arrhythmias, and incomplete congenital stationary night blindness, and are reviewed elsewhere (for details on L-type channelopathies please see the reviews [55,56]). Historically, TTCCs were implicated in several neurological conditions. It is now well known that TTCCs are implicated in several other pathological conditions including epilepsy, inflammatory pain, Parkinsonian tremors, and heart failure [57,58,59].

Interestingly, in the past decade, VGCCs have been implicated in the pathology of various cancers and are being explored as potential targets [60,61,62]. There is some evidence to suggest a causal role of VGCCs in various cancers, however, it needs more definite evidence. Complete details of the occurrence and expression of VGCCs in 19 types of cancer from human patients are available in online databases such as cBioPortal and oncomine [61,63,64].

Lately, some studies have demonstrated the causal/therapeutic roles of TTCCs and LTCCs in the pathology of breast cancer [65,66,67,68]. Since the hormone estrogen plays an important role in the pathology of breast cancer, we found it compelling to understand the interactions between estrogen and VGCCs especially when studies have reported that estrogen can modulate VGCCs in other tissues as well. In this review, we describe what is known about the estrogenic regulation of VGCCs in general and LTCCs and TTCCs in particular. We conclude with insights on the gaps in the knowledge and perhaps what we need to know if we were to pursue estrogen regulation of VGCCs as a therapeutic target.

### 1.2. Estrogen-Estrogen Receptor Signaling

The steroid hormone estrogen is involved in varied physiological functions such as regulation of the central nervous system, immune and cardiovascular system homeostasis, epithelial cell proliferation in mammary glands and endometrium [69,70], cholesterol mobilization, control of inflammation, sperm maturation, regulation of gene expression, etc. [71,72,73,74]. The predominant intracellular estrogen is 17β-estradiol (estrogen). Two classical estrogen receptor (ER) subtypes have been reported which are ER alpha (ERα, encoded by ESR1) and ER beta (ERβ, encoded by ESR2), which are expressed abundantly in tissues such as bone, brain, breast, liver, ovary and uterus [75]. The hormone–receptor complex (estrogen–ER complex) binds to the specific sequence in DNA called as estrogen response element (ERE) to regulate gene expression in an ERE-dependent manner. Estrogen can also function in an ERE-independent manner that involves binding of the estrogen–ER complex to DNA transcriptional factors such as AP1, c-Fos, etc., to further stimulate gene expression [76]. In addition to the ligand (estrogen)-induced activation of ERs, ligand-independent signaling has also been described where epidermal growth factor receptor (EGFR) and insulin-like growth factor receptors can activate protein kinase cascade which phosphorylates and activate ERs [77].

The two ER subtypes are functionally distinct from each other and ERβ opposes the activation of ERα [78,79]. While there are abundant studies on the role of ERα (for recent reviews please refer to [80,81]), studies on ERβ are limited. Deregulation of estrogen and signaling via ER is associated with malignancies [82,83,84]. In particular, estrogen not only plays an important part in the physiological functioning of breast tissue, but also plays a causal role in the manifestation of breast cancer, as evidenced by the use of ER blockers (ERBs) as therapeutic agents in a few subtypes of breast cancer which are termed as receptor-positive subtypes (for breast cancer overview please refer [85,86]). In these receptor-positive subtypes of breast cancer, ERα is abundant. Not only it is abundant, but it has a causal role and thus, ERBs are used as therapeutic drugs [87,88,89]. The role of ERβ has been disputed and a dual proliferative/anti-proliferative role has been suggested in cancer [90,91]. There could be several possible reasons for the dual proliferative/anti-proliferative role of ERβ such as 1) there could be differential expression or actions of ERβ isoforms-ERβ1, ERβ2, and ERβ5. So far, only ERβ1 (full-length protein) has been investigated. This is also due to a lack of isoform-specific antibodies for ERβ. Perhaps investigating other isoforms may lead to a better understanding of the action of ERβ in cancer. 2)EGFR can modulate ERβ’s growth-promoting effects since it is also active in triple-negative breast cancer (TNBC) cell lines [92,93,94], therefore any change in the expression and activity of EGFR may lead to modulation of ERβ signaling. 3)Tumor protein p53 (TP53) status as wild type or mutant could define the role of ERβ [95]. It was observed that ERβ interaction with wild-type TP53 caused a pro-proliferative effect whereas ERβ interaction with mutant TP53 showed anti-proliferative effects. Therefore, more studies are perhaps needed to understand the exact role of ERβ in physiology and pathology.

Apart from the estrogen produced in vivo, we are also exposed to environmental estrogens frequently. Environmental estrogens comprise xenoestrogens which are synthetic chemicals and phytoestrogens which are of plant origin. Both xenoestrogens and phytoestrogens are structurally similar to estrogen and may mimic the function of estrogen in vivo, thus higher exposure to such chemicals may have detrimental effects. In this study, we also provide evidence that suggest that environmental estrogens can modulate VGCCs to mediate physiological or potentially toxic effects. Inhibition or upregulation of VGCCs also depended upon the type of environmental estrogen.

## 2. Ion Channel Regulation by Estrogen

As mentioned briefly in the previous section, estrogen binds to the ER forming an estrogen–ER complex which then translocates to the nucleus and binds to ERE to either induce or repress the expression of genes encoding the ion channels. This forms the transcriptional pathway (genomic) of ion channel regulation by estrogen [96]. Non-transcriptional (non-genomic) pathway of ion channel regulation by estrogen involves binding of estrogen to the membrane receptors (G-protein coupled receptors, insulin-like growth factor receptor, etc.), which activates downstream signaling cascades targeting the ion channels or by activation of cytoplasmic second messengers [96].

Regulation of ion channels by estrogen was first reported in the cardiovascular system where estrogen indirectly increased current through a calcium-activated potassium channel [97]. Afterward, numerous studies reported the regulation of ion channels by estrogen in a variety of cell types including the brain, breast, heart, muscle, and kidney [98,99,100]. This includes sodium channels, calcium channels, chloride channels, etc. The action of estrogen on ion channels in physiological and cancer conditions is recently reviewed [101,102]. Very recently, Jiao and colleagues have reviewed the effects of estrogen on calcium handling proteins in cardiac myocytes as they relate to cardiovascular disorders [103].

In this review, we will focus on the modulation of VGCC expression and activity by estrogen in physiological or pathological conditions either in a genomic or a non-genomic manner.

### 2.1. Estrogenic Regulation of VGCCs in Physiological Conditions

Due to the widespread expression of ERs and VGCCs across several tissues, estrogen regulation of VGCCs has been described in various cell types.

#### 2.1.1. Estrogen Affects VGCC Current in Cardiovascular Tissues

##### Estrogen-Mediated Inhibition of VGCCs in Cardiac Tissues and Mechanisms Therein

In the early research, it was clearly observed that estrogen affects contractile responses in cardiovascular cells such as smooth muscle cells [104,105]. With the discovery of functional ERs in the vascular smooth muscle cells and cardiac myocytes, researchers began to investigate the influence of estrogen on VGCCs in cardiomyocytes in light of the fact that estrogen can modulate voltage-gated ion channels. Early in vivo studies demonstrated a vasodilatory effect of estrogen and combined with the in vitro data, it was suggested that estrogen at physiological doses exhibited a vasodilating effect in the vascular smooth muscle cells of Sprague Dawley rats by inhibiting currents through VGCCs [106]. Later, since LTCCs are predominantly involved in the excitation–contraction coupling in cardiac cells and estrogen affects contractile responses, a plethora of in vitro and ex vivo studies have contributed to the understanding of estrogenic regulation of LTCCs.

Inhibition of voltage-gated calcium influx by acute application of estrogen was shown very early in a variety of cardiac cells and tissues such as isolated adult ventricular myocytes of guinea pigs [107,108,109], isolated adult ventricular myocytes of rats [109,110], rat aorta [111], rabbit coronary arteries [112], rabbit arterial tissue [113], isolated atrial myocytes from human hearts [109] and vascular smooth muscles of rats and rabbits [114]. In most of these studies, inhibition of inward voltage-gated calcium current by estrogen was achieved within minutes (with a time constant of 3–4 s) and IC_50_ values obtained in the presence and absence of ER antagonist ICI 182780 were similar, indicating that antagonizing ERs did not alter estrogen effect; therefore, perhaps the mechanism of action of estrogen did not involve classical hormonal receptor activation [111]. This line of thought was further supported by the work of Salom and colleagues who showed that the endothelium-independent acute relaxant effects of estrogen in the rabbit carotid artery were mimicked by the LTCC blocker, nicardipine [115]. In addition, treatment with ER antagonist ICI 182780 and cycloheximide, a protein synthesis inhibitor did not alter the estrogen-induced relaxant effect. This indicated that estrogen elicited a non-genomic response and the relaxant effect was due to the inhibition of voltage-gated calcium influx through LTCCs [115]. Since the discovery of nuclear ERs [116] in cardiac myocytes, it was suggested that genomic mechanisms may also be operating. It is also important to note that in most of these studies, inhibition of VGCCs occurred at high doses of estrogen, the relevance of which may be limited in vivo, but these studies provided important information regarding modulation of VGCCs by estrogen and that VGCCs in cardiac cells are important targets for estrogen. By this time the identity of VGCCs in the cardiovascular system was also discovered. A7r5 cell line derived from mouse aortic smooth muscle revealed the presence of HVA LTCCs and LVA TTCCs [117,118,119] in these cells indicating that estrogen maybe acting on these two VGCCs in the cardiac cells to inhibit voltage-gated calcium influx. Indeed, whole-cell patch-clamp experiments in A7r5 cells revealed that estrogen can inhibit both LTCCs and TTCCs in these cells [120]. This in vitro data from the smooth muscle cell line corroborates well with the in vivo, in vitro and ex vivo studies in cardiac cells and tissues showing inhibition of LTCCs by estrogen in a non-genomic manner. In order to gain mechanistic insights into the non-genomic regulation of LTCCs by estrogen, Ullrich and colleagues hypothesized that estrogen may directly interact with the cardiac LTCCs [121]. Using HEK-293 cells, which do not express endogenous ERs or cardiac LTCCs, they demonstrated that estrogen rapidly reduced the calcium current in the LTCC (Ca_V_1.2)-transfected HEK-293 cells and caused a shift in the voltage-dependent activation towards more negative potentials [121]. The inhibitory action of estrogen occurred more rapidly at higher stimulation frequencies exhibiting an accumulation of channel block. This is the only report indicating a direct interaction between estrogen and LTCCs using a heterologous expression system, however, it provided mechanistic insights into the non-genomic regulation of LTCCs by estrogen [121]. Further mechanistic insights into the non-genomic regulation of LTCCs by estrogen revealed the involvement of a G-protein coupled receptor for estrogen (GPER1, 7-transmembrane GPCR that responds to estrogen with rapid cellular signaling), where GPER1 agonist G-1 and estrogen inhibited the KCl-evoked increase in intracellular calcium concentration in the vascular smooth muscle cell line A7r5. This increase in intracellular calcium concentration was also inhibited by the LTCC blocker nifedipine indicating that GPER1 (also called GPR30) regulates calcium influx via LTCCs and this could be the mechanism through which GPER1 controls blood pressure [122]. GPER activation also prevented the β adrenoceptor agonist-(isoproterenol) induced potentiation of calcium signals by decreasing the PKA-dependent phosphorylation of Ca_V_1.2 LTCC indicating that GPER functions as an intrinsic component in the β-adrenoceptor-mediated signaling in cardiomyocytes, possibly mediating a protective feedback mechanism to control the heart against calcium overload [123]. β-adrenoceptor-mediated signaling in cardiomyocytes is indispensable to fight and flight response, which temporarily increases cardiac contractility. Non-genomic mechanisms of estrogen inhibition of VGCCs are also described for TTCCs where long-term (24 h) estrogen treatment in neonatal cardiomyocytes reduced the mRNA expression of TTCC isoform Ca_V_3.2 [124]. Signaling through cardiac homeobox transcription factor Csx/Nkx2.5 along with extracellularly regulated kinase (ERK-1/2, 5) led to the downregulation of TTCC current by estrogen [124]. This ER-independent action was confirmed in the heterologous expression system where incubation of HEK-Ca_V_3.2 transfected cells with different concentrations of estrogen significantly reduced the TTCC current. Since HEK cells do not express ER subtypes, these results provided clarity that the modulation of TTCCs by estrogen was independent of the ER signaling pathway and thus also non-genomic, similar to LTCCs [124].

Apart from non-genomic mechanisms of estrogen action on VGCCs, early evidence also indicated genomic mechanisms of estrogen regulation of LTCCs. ER-deficient mice revealed increased expression and activity of LTCCs as determined by increased calcium channel antagonist isradipine binding and increased LTCC current in the ventricular cardiomyocytes dissociated from the ER-deficient mice [125]. This indicated that estrogen also regulates the expression and activity of LTCCs via the ERs. Recent evidence indicated that prolonged treatment (24 h) with nM concentration of estrogen not only inhibited the LTCC current but also reduced the protein expression of LTCC Ca_V_1.2 [126]. The addition of an ER antagonist IC1 182780 eliminated the effect of estrogen on LTCC expression. This was also the first study on arterial smooth muscle cells of female Yorkshire pigs demonstrating that estrogen binds to ERα/ERβ to alter the post-transcriptional regulation of the LTCC Ca_V_1.2 [126]. The effects of estrogen on VGCCs in cardiovascular tissues are listed in Table 2.

##### Estrogen-Mediated Upregulation of VGCCs in Cardiac Tissues

Contrary to the early research showing inhibition of VGCCs by estrogen, studies in the last decade showed evidence of upregulation of LTCC Ca_V_1.2 by estrogen and phytoestrogen quercetin [132]. In ventricular cardiac myocytes from the base of the female rabbit, estrogen upregulated LTCC Ca_V_1.2 current in an ERα-dependent manner. This upregulation was regional and not observed in the endocardium, apex, or cardiac myocytes from the base of the male rabbits [127]. In these cells, estrogen treatment also increased the sodium–calcium exchanger (NCX1) protein level and current but not in the endocardium, apex, or male base cardiac myocytes. Thus, the higher calcium influx via upregulated LTCCs was balanced by the higher efflux mediated by the upregulated NCX1 [128]. This provided new insights and implicated ERs as potential targets in cases where females are more prone to lethal arrhythmias. Later, Papp and colleagues examined whether the estrogenic regulation of LTCC current in female rabbit hearts applies to the human heart as well [129]. Analysis of protein expression in the post-mortem human left ventricular tissue samples revealed higher Ca_V_1.2 and NCX1 in women at the base rather than apex of epicardium as compared to males or postmenopausal women indicating that regional differences in LTCC regulation may be attributed to estrogen [129]. In support of this, increased LTCC mRNA and protein levels were observed in cardiomyocytes derived from female human induced pluripotent stem cells derived cardiac myocytes treated with estrogen (iPS-CMS) [129]. Upregulation of calcium influx mediated by LTCCs can also occur via estrogen action on GPER (GPR30) and also by activating PI3K, Akt, and cAMP-response element binding protein (CREB) signaling in a non-genomic manner [130,131]. Direct evidence of GPR30 involvement came from the ability of GPR30 antagonist G15 to abolish the estrogen-mediated upregulation of LTCC mRNA and protein levels in ovariectomized estrogen-treated female mice with no effect of ER antagonist ICI 182780 [131]. Many times, the genomic and non-genomic mechanisms operate simultaneously. Evidence that supports the possibility of synergism is the adjacent/overlapping binding sites for p-CREB and ERα in the promoter regions of the CACNA1C gene (human–rabbit–rat) encoding Ca_V_1.2 [130].

Taken together, it is clear that estrogen-mediated increase in VGCC protein expression or current occurred at lower (nM) concentrations of estrogen while the inhibition of VGCCs usually occurred at higher doses of estrogen during acute treatment. This indicates that rather than inhibition or activation, estrogen regulates VGCCs depending on the amount and duration of estrogen to fine-tune cellular processes. Most interestingly, estrogenic regulation of VGCCs has also highlighted the apex-base heterogeneities where regulation of VGCCs by estrogen is different in the apex versus base of the heart in a gender-specific manner. Such apex-base heterogeneity has been described structurally in the heart, where base cardiomyocytes are much more organized with an increased number of specialized microdomains such as T-tubules and caveolae [133]. The higher number of caveolae in the base cardiomyocytes provides tighter control of β-adrenergic stimulation in these cells [133]. A subset of LTCCs also reside in the caveolae microdomains [134]; therefore, it would be certainly pertinent to determine if the estrogenic regulation of LTCCs is also dependent on the structural microdomains. Since there are gender-specific differences in the structural microdomains such as T-tubules and caveolae, this could redefine the mechanistic basis of estrogen regulation of VGCCs in the heart and could potentially lay the background for gender-specific treatment of cardiovascular disorders in light of the fact that there is a gender-specific risk of arrhythmia [135,136,137]. Notably, structural domains such as caveolae are not restricted to the heart, in fact, caveolae are a subset of lipid rafts that are rich in caveolin protein and are present ubiquitously. In certain cells, estrogen signaling was observed to be dependent on the lipid rafts and expression of caveolin, where cholesterol depletion abolished estrogen-dependent effects such as estrogen-dependent platelet aggregation [138]. Mechanistically, palmitoylation of ERα initiated ERα association with the plasma membrane and interaction with caveolin thus contributing to its non-genomic effects including activation of signaling pathways (ERK and AKT activation, etc.). Thus, it seems that ER can be part of a signalosome on the plasma membrane where many proteins are clustered. VGCCs and other ion channels are also clustered in signalosomes on the membrane where they are localized in caveolae [139]. However, the involvement of lipid rafts and caveolae microdomains in the regulation of VGCC activity or expression by estrogen remains elusive. Whether the presence or absence of ERs in caveolae could govern inhibition or upregulation of VGCCs remains to be investigated.

#### 2.1.2. Estrogen Affects VGCC Currents in Neuronal Tissues

##### Estrogen-Mediated Inhibition of VGCCs in Neuronal Tissues and Mechanisms Therein

The importance of VGCCs in neuronal excitability and widespread expression of ERs in neuronal cells and tissues led to the investigation of estrogenic regulation of VGCCs in neurons as well. In the rat neostriatal neurons, estrogen showed a reduction of barium entry via calcium channels in the acutely dissociated as well as cultured neurons [140]. Nifedipine, an LTCC antagonist exhibited a strong occlusion of the estrogen response compared to other calcium channel blockers, suggesting that estrogen primarily modulated LTCCs in rat neostriatal neurons [140]. The modulation was dose-dependent, steroid as well as sex-specific and it appeared to occur via membrane ERs [140]. In the acutely dissociated rat dorsal root ganglion sensory (DRG) neurons estrogen also showed sex-specific modulation of HVA L- and N-type VGCCs where, estrogen-BSA, a membrane-impermeable conjugate showed inhibition of peak HVA currents suggesting that these effects also occurred in a non-genomic manner through membrane receptors [141]. This inhibitory effect of estrogen on the HVA currents was higher in female rat DRG neurons than in males [141]. These results were found to be consistent with estrogen inhibition of calcium influx and VGCC current observed in the cardiovascular tissue as described in the previous section. In another interesting observation, estrogen treatment reversed the age-related increase in LTCC current in the zipper slice exposing the hippocampal pyramidal neurons which correlated with decreased mRNA expression of Ca_V_1.2 LTCC channel in aged female rats treated with estrogen [142]. This study is in agreement with other in vitro data documenting the inhibition of LTCCs by estrogen. In the cultured hippocampal cells from rats, acute application of estrogen inhibited the γ-aminobutyric acid (GABA), and N-methyl D-aspartate acid (NMDA), inducing an increase in the intracellular calcium concentration mainly by inhibition of LTCCs. Since treatment with ER antagonist tamoxifen did not interfere with the estrogen effect, it indicated a non-genomic mechanism of action [143]. Studies on the PC12-neuroendocrine cells revealed that estrogen inhibited the rise in cytosolic free calcium concentration by inhibition of L-and N-type VGCCs. Mechanistically, in these cells, treatment with protein synthesis inhibitor (cycloheximide), transcription inhibitor (actinomycin D), and G-protein sensitive pertussis/cholera toxins did not affect the estrogen action ruling out the possibility of genomic or G-protein mediated mechanism of action again indicating non-genomic mechanisms [144]. Estrogen inhibition of HVA calcium channel current in cultured rat cortical neurons was observed through voltage-clamp experiments [145]. In these experiments, ER antagonist ICI 182780 could not prevent the inhibitory action of estrogen on the HVA calcium current indicating an ER-independent mechanism. However, the inhibitory action of estrogen on the HVA calcium current could be prevented by protein kinase A (PKA) or protein kinase C (PKC) antagonists indicating that estrogen modulation of the HVA calcium current depended on PKA/PKC signaling pathway rather than ERs [145]. However, this mechanism was contradicted by the study of Sanchez and colleagues, where treatment of rat cortical neurons with either ER antagonists or inhibitors of PKA/PKC did not affect the estrogen-mediated inhibition of HVA LTCCs suggesting that more than one mechanism may be operating in neuronal tissues [146]. In addition to endogenous estrogen, environmental xenoestrogens nonylphenol and bisphenol A (BPA) also inhibited the HVA total calcium current and LTCCs, respectively, in the GH3 pituitary cells [147,148], both HVA and LVA currents in mouse DRG neurons and recombinant human R-type calcium channels expressed in HEK293 cells [147]. Xenoestrogens such as hydroxylated polybrominated diphenyl ethers (PBDEs) and nondioxin-like polychlorinated biphenyls (NDL-PDBs) inhibited the depolarization-evoked basal intracellular calcium concentration in rat pheochromocytoma (PC12) neuronal cell line. This inhibition of calcium influx was mediated by their action on VGCCs [149,150]. While all these studies demonstrated an inhibitory action of estrogen on the HVA calcium currents, augmentation of VGCCs, and in particular, LVA TTCCs, by estrogen are also reported in the neurons as described below.

##### Estrogen-Mediated Upregulation of VGCCs in Neuronal Tissues

Extremely low concentrations of estrogen could acutely potentiate VGCCs in primary hippocampal cultures, hippocampal slices, and HEK-293 cells transfected with LTCCs in an ER-independent manner [151]. Interestingly, using cell binding assays, the authors showed that estrogen can directly bind to LTCCs [151]. In addition, the effects of estrogen were significantly attenuated in a mutant, dihydropyridine-insensitive LTCC, indicating not only the direct interaction of estrogen with LTCCs but also that estrogen and dihydropyridines may share a common motif for interaction with LTCCs [151]. In vitro experiments evaluating estrogen effects on the ovariectomized mice GnRH neurons revealed that estrogen increased HVA calcium currents through L- and R-type VGCCs and this potentiation was initiated by binding of estrogen to ERβ and GPR30 [152]. In GnRH-producing GT1-7 neuronal cells, estrogen differentially modulated C5a-evoked calcium influx via LTCC depending on the expression of ERα and ERβ [153]. 24 h application of estrogen and ERα agonist, 4, 4′, 4″-(4-Propyl-[1*H*]-pyrazole-1, 3, 5-triyl) trisphenol (PPT) increased the calcium influx evoked by PL-37 MAP (C5aR agonist peptide) while ERβ significantly reduced the calcium influx. In contrast, short-term application (8 min) of ER agonist did not affect the calcium influx indicating that the estrogen effect on the calcium influx through LTCCs was through genomic mechanisms and was also dependent on ER isoforms [153]. Further, Sedej and colleagues provided insights into the VGCC modulation by estrogen in mouse melanotrophs in pituitary slices [154]. They observed LTCC expression to be dominant and upregulated by increased estrogen levels in newborn melanotrophs. On the mechanistic level, acute perfusion of estrogen in pituitary slices failed to detect the considerable changes in the calcium amplitude or kinetics, suggesting that estrogen modulated LTCCs at the genomic level rather than a non-genomic level [154].

Not only LTCCs, but estrogen treatment of female guinea pigs also revealed higher mRNA expression of TTCC isoform Ca_V_3.1 in the hypothalamic and arcuate neurons [155]. Functional analysis of calcium currents from brain slices revealed that estrogen increased TTCC current in arcuate neurons during the rebound burst firing which correlated with the higher expression levels [155]. This was a significant study demonstrating an increase in TTCC mRNA expression as well as a current influx in the central nervous system neurons upon estrogen treatment unlike most other invitro studies that have reported estrogen-mediated inhibition of calcium channels. Using ERαKO (ERα knockout) and KIKO (ERα knockin/knockout-lacking functional ERE binding domain), Yang JA and colleagues also showed that TTCC Ca_V_3.1 mRNA expression was upregulated in the arcuate nucleus and this increase was found to be more than two-fold in wild type (WT) but not in ERαKO and KIKO suggesting an ERα-mediated and ERE-dependent regulation of TTCCs [156]. Later, estrogen augmentation of LVA calcium currents was also observed in the hypothalamic neurons by others [157]. Estrogen treatment enhanced the magnitude of LVA calcium current in the hypothalamic neurons in the absence of phenylephrine, an α_1_adrenergic agonist. In contrast, in its presence, augmentation of HVA calcium currents mediated by N- and L-type VGCCs was observed [157]. This reiterated crosstalk between adrenergic and estrogen signaling in the brain like heart but the overall mechanism of action remains unclear at the moment. The mRNA expression level of all three TTCC isoforms was also found to be significantly increased in the gonadotropin-releasing hormone (GnRH) neurons obtained from the estrogen-treated mice [158]. It was intriguing to note that, while mRNA expression was increased during the morning, the mRNA levels of TTCCs reduced during the afternoon [158]. To understand the mechanism involved in the estrogenic regulation of these channels, mice were treated with membrane ER agonist STX, a non-steroidal diphenylacrylamide compound that specifically binds to the plasma membrane ER. In females treated with STX, mRNA expression of TTCC isoform Ca_V_3.3 was higher, indicating a possibility that estrogen regulates TTCCs in GnRH neurons via membrane ERs in an isoform-specific manner [158]. Sun J and colleagues further investigated the effects of estrogen on VGCCs in GnRH neurons in brain slices from ovariectomized mice (OVX) and OVX mice treated with estrogen (OVX + E) [152]. They observed that LVA calcium currents were not altered by the estrogen treatment. On the other hand, HVA calcium currents varied with daylight time in OVX + E mice. Their results, though slightly different from Zhang and colleagues [158], also revealed diurnal changes, but in their studies, these diurnal changes were associated with L-and N-type HVA currents and not TTCCs as observed by Zhang and colleagues [152]. In an attempt to further explore the mechanism of isoform-specific regulation of TTCCs by estrogen, ERα and ERβ knockout mice (αERKO and βERKO) were used [159]. In this study, Bosch MA et al. reported that estrogen treatment significantly increased the mRNA expression of Ca_V_3.1 in the WT and βERKO mice in the arcuate nucleus while Ca_V_3.2 mRNA upregulation was observed only in WT and not in αERKO and βERKO. Moreover, Ca_V_3.3 mRNA expression remained unaltered in the hypothalamic regions. In the pituitary, Ca_V_3.1 mRNA expression was increased whereas Ca_V_3.2 and Ca_V_3.3 mRNA expression was decreased in WT and βERKO mice. In contrast, estrogenic effects were completely lost in αERKO animals thereby indicating that estrogen regulation of Ca_V_3.1 mRNA depended on ERα and Ca_V_3.2 mRNA depended on both ERα and ERβ in the hypothalamic region. Whereas, in the pituitary, estrogen effects on TCCC isoforms depended on ERα only [159]. Comparative gene expression microarray using RNA extracted from the pituitaries of metestrous (low estrogen) and proestrus (high estrogen) stage mice, as well as from ovariectomized WT and ERα knockout mice treated with estrogen or vehicle, revealed that estrogen upregulated P/Q type calcium channel mRNA CACNA1A (Ca_V_2.1) and TTCC (Ca_V_3.1) mRNA in the pituitary and that these channels were dependent on ERα for their pituitary expression [160]. This is in accord with the previous study reported by Bosch and colleagues which also demonstrated that estrogenic modulation of TTCC in the pituitary is ERα-dependent [159]. In retinal cells, estrogen-mediated retinal protection by increasing intracellular calcium concentration by calcium influx through LTCCs and PI3K pathway [161]. An increase in the calcium influx via action on L- and TTCCs in GnRH-expressing GT1-7 cells was also reported for xenoestrogen BPA, unlike its inhibitory effect on VGCCs in other tissues [162].

Table 3 lists the estrogenic modulation of VGCCs in the neuronal tissues. Overall, more studies reported inhibition of HVA LTCCs upon estrogen treatment in neuronal cells, however, TTCCs were mostly upregulated upon estrogen treatment. This indicates differential modulation of VGCCs and their isoforms by estrogen. Estrogen signaling seems complex and at the moment it is difficult to conclude what determines genomic or non-genomic mechanisms. Most importantly, the identity of players in non-genomic mechanisms needs to be further explored.

#### 2.1.3. Estrogen Modulates VGCCs in Spermatogenic Cells

TTCCs present in the plasma membrane of spermatogenic cells are believed to be the major contributor of calcium influx in these cells [163]. Based on the fact that estrogen is essential for sperm capacitation and acrosome reaction, the effect of estrogen on VGCCs was studied in spermatogenic cells [164]. Estrogen inhibited TTCC current in spermatogenic cells in a voltage-dependent manner, as seen in cardiovascular and neuronal tissues [164]. Not only estrogen but also raloxifene, a selective estrogen receptor modulator (SERM) also inhibited TTCC current in the mouse spermatocytes [165]. In this study, since the presence of ER antagonist ICI 182780 could not attenuate inhibition and the raloxifene effect occurred within 5 min of exposure, it was speculated that non-genomic mechanisms could be operating. However, raloxifene also decreased the mRNA expression of TTCCs (Ca_V_3.2 and Ca_V_3.3, not Ca_V_3.1) indicating that genomic and non-genomic mechanisms operate hand-in-hand depending on the duration and concentration of estrogen or ER modulators [165].

#### 2.1.4. Estrogen Modulates VGCCs in Uterine Cells

VGCCs and ERs are important players in uterine physiology and pathophysiology. Twenty-four-hour estrogen treatment at different concentrations downregulated all three TTCC isoforms Ca_V_3.1, Ca_V_3.2, and Ca_V_3.3 only at the highest concentration of 1 µM estrogen in the telocytes from human pregnant uterine myometrial cultures [166]. Estrogen treatment had no effect on the mRNA expression of LTCC Ca_V_1.2 [166]. Telocytes (earlier called cajal-like cells) are interstitial cells characterized by long, thin cytoplasmic projections called telepods. They are present in many tissues and organs including the female reproductive system and are known to regulate tissue homeostasis including calcium homeostasis. In the uterine myometrium, telocytes act as hormone sensors and can change their morphology and function during the endometrial cycle as well as the pregnancy period [167]. Calcium signaling plays an essential role in the contractility of telocytes. Interestingly, estrogen treatment also downregulated the mRNA expression levels of ERα, ERβ, and GPR30 indicating a feedback phenomenon. In patchclamp recordings, estrogen inhibited HVA LTCC current during acute exposure [166]. The effect of estrogen on TTCC current was not determined in the study, however, it would be rather more relevant to determine if TTCC current can be observed in telocytes and if it can be inhibited by estrogen since the effect of estrogen was more prominent on the mRNA expression of TTCCs. The most interesting finding of this study was the isoform-specific modulation of TTCCs during pregnancy since estrogen is one of the major pregnancy hormones. Pregnancy significantly upregulated Ca_V_3.1 mRNA, however, it downregulated other isoforms Ca_V_3.2, Ca_V_3.3, and also ESR1 and ESR2. It would be thus interesting to determine isoform-specific regulation of TTCCs by estrogen in more physiological settings. This also indicated that estrogen has complex signaling and more physiological assays/experimental models are required to assess the effect of estrogen on VGCCs than isolated cells or heterologous expression systems.

#### 2.1.5. Estrogen Modulates VGCCs in Immune Cells

In macrophage RAW-264.7 cells, calcium influx through LTCCs is partially responsible for the delayed estrogen signaling that is observed [168]. In these cells, different doses of estrogen–BSA conjugates could not alter the calcium influx, whereas, unconjugated estrogen stimulated the calcium influx indicating that the delayed estrogen signaling was mediated by intracellular receptors, although details on the specific type of intracellular receptor involved in this estrogen-mediated effect on calcium influx remain unanswered [168]. A similar effect was observed in the mouse bone marrow-derived macrophages where estrogen induced a rise in the intracellular free calcium via extracellular calcium influx through TTCCs [169]. This estrogen-triggered calcium influx attenuated lipopolysaccharide (LPS)-stimulated tumor necrosis factor alpha (TNF-α) production via blockade of the p38 MAPK pathway. Contrary to macrophages, in bone marrow cells, estrogen–BSA conjugates also caused calcium influx. In addition, the inhibitory action of estrogen on calcium influx was not sensitive to intracellular ER inhibitors such as ICI 182780 and tamoxifen thus indicating a possibility of a membrane receptor-mediated non-genomic pathway.

### 2.2. Estrogenic Regulation of VGCCs in Pathological Conditions

Due to the regulation of VGCCs by estrogen in physiological conditions, it was intriguing to evaluate how this interaction could be different or contribute to pathological conditions. A handful of studies have investigated the interaction between estrogen and VGCCs in pathophysiology.

#### 2.2.1. Altered Cardiac Function

Xenoestrogens and phytoestrogens have the ability to alter cardiac function and thus have the potential to cause cardiotoxicity. Non-genomic regulation of LTCCs is described for xenoestrogens such as alkylphenol detergents (octylphenol and lipid soluble organochloride pesticides such as dichlorodiphenyltrichloroethane), nonylphenol and BPA, which inhibited calcium influx through LTCCs in A7r5 vascular smooth muscle cells [170], female Sprague Dawley rat heart [171] and mouse cardiac myocytes [147]. Acute exposure to BPA promoted arrhythmogenesis in the female rat heart which involved alterations in calcium handling proteins. BPA showed monotonic effect on the individual processes of rat cardiomyocyte calcium handling proteins, where, at lower concentrations, BPA showed a rapid increase in the calcium transient and consistent stimulation of LTCC currents in an ERβ-mediated manner [172]. This suggests that alterations in the myocyte’s calcium channels underlie the proarrhythmic action of BPA. Phytoestrogen yangambin (a lignan isolated from Ocotea duckei Vattimo (Lauraceae)), induced hypotension in rats while in the isolated rat atria, negative ionotropic, and chronotropic effects were observed. Using in vitro and in vivo approaches, it was revealed that yangambin induced hypotension by peripheral vasodilation that involved inhibition of calcium influx through VGCCs [173]. This explains the vasorelaxant actions of environmental estrogenic pollutants, however, since environmental estrogens can accumulate in humans it may have consequences for people with cardiovascular disorders and already taking drugs to modulate the vascular tone.

#### 2.2.2. Neurodegeneration

Dysfunction of LTCCs is seen in pathological conditions such as Alzheimer’s disease (AD) and Parkinson’s [174]. On the contrary, estrogen is shown to be neuroprotective and its deficiency is implicated in AD-like pathologies [175,176]. In the ovariectomized mice OVXAPP/PS (a mouse model of AD), the basal level of HVA Ca_V_1.2 expression was increased in the hippocampus and cortex, which could be inhibited by selective ERα agonist PPT treatment and accompanied by improved cognitive functions [177]. The peak calcium current density as determined by whole-cell patchclamp experiments on cortical neurons was significantly reduced upon estrogen treatment (24 h) and non-selective ER antagonist ICI 182780 attenuated the effect [177]. In addition, PPT treatment significantly reduced the Ca_V_1.2 protein expression and current density indicating that estrogen modulated Ca_V_1.2 expression through ERα to provide neuroprotection. Mechanistic studies using mouse hippocampal cell line HT22 and human neuroblastoma cell line SH-SY5Y revealed a possible association of E3 ligase Mdm2 (double minute 2 protein) with Ca_V_1.2 upon activation of ERα. This process of Ca_V_1.2 regulation involved K29-linked ubiquitin chains and PEST sequence in Ca_V_1.2 which may have provided a signal for ubiquitination/proteasomal degradation [177]. In ventral spinal motoneuron and neuroblastoma hybrid cell line VSC 4.1, estrogen showed a protective role by limiting glutamate-induced cell death by inhibiting calcium influx through LTCCs [178]. This attenuated calcium influx also inhibited the activity of pro-apoptotic proteases caspase-3 and calpain thus providing protection [178]. In these cells, treatment with LTCC agonist FPL 64179 increased intracellular free calcium and cell death whereas estrogen inhibited both the effects [178]. Overall, estrogen regulation of calcium influx through VGCCs provided a mechanistic basis for the neuroprotection observed upon estrogen presence/treatment. This may provide a therapeutic opportunity targeting VGCCs by estrogen for neuroprotection.

#### 2.2.3. Carcinogenesis

Estrogen has been strongly linked to the manifestation of breast and gynecological cancers. For detailed reviews please refer [179,180,181]. Altered gene expression of VGCCs is often seen in several cancers [182,183]. Particularly in cancers that involve estrogen, the interaction between VGCCs and estrogen was investigated. Overexpression of LTCC isoform Ca_V_1.3 was observed in endometrial cancer and atypical hyperplasia where shRNA knockdown of Ca_V_1.3 inhibited migration and proliferation in the ER-positive endometrial cancer (Ishikawa) cell line [184]. When Ishikawa cells were subjected to estrogen stimulation using either estrogen or estrogen-BSA, upregulation of Ca_V_1.3 expression was observed within 30 min of estrogen treatment providing the mechanistic basis for estrogen-induced pathology in ER-positive cancers [184]. Since then, a few studies have investigated the regulation of LTCCs and TTCCs by estrogen in breast cancer using cell lines. Estrogen significantly upregulated LTCC Ca_V_1.3 protein expression in a dose and time-dependent manner in the MCF-7 luminal-type ER-positive breast cancer cell line which was suppressed upon treatment with GPCR antagonist PTX revealing a non-genomic mechanism of action of estrogen in breast cancer [185]. This study indicates that perhaps estrogen facilitates tumor progression by increasing calcium influx through VGCCs and that they could be a target in breast cancer as suggested by others [61,65,87].

While investigating the role of calcium pumps and proteins in breast cancer calcium signaling, Pera et al. observed that a high level of Ca_V_3.2 expression in luminal type ER-positive breast cancer co-occurred with an increased level of ESR1 in patient samples from the TCGA (The Cancer Genome Atlas) database [186]. However, overexpression of Ca_V_3.2 failed to increase the expression of ESR1 in HER2-positive SKBR3 cell line (luminal type) suggesting that overexpression of Ca_V_3.2 perhaps is not the driving force for the pathogenesis of luminal type breast cancer. Survival analysis of patient data suggested that Ca_V_3.2 could be a potential differential biomarker in breast cancer subtypes where the overexpression of Ca_V_3.2 was associated with poor disease outcomes in patients with ER-positive breast cancer, while Ca_V_3.2 expression was positively correlated with patient survival after chemotherapy in patients with HER2 positive breast cancer [186]. Since breast cancer subtypes differ in the expression of ERs, we speculate that calcium signaling involving TTCCs and ERs could play a role in the differential outcome. Additionally, whether overexpression of ESR1 can lead to overexpression of TTCCs in breast cancer needs to be investigated, since plenty of evidence indicates that estrogen/ER can modulate the expression of VGCCs and TTCCs and not vice-versa.

Recently, subtype-specific alteration of TTCCs and ER isoforms was shown in breast cancer subtypes [187]. Some of the alterations in TTCCs and ER isoforms co-occurred in breast cancer subtypes and therefore targeting them together could be beneficial [187]. Perhaps, it would be interesting to determine the regulation of TTCCs by estrogen in breast cancer. To the best of our knowledge, there are no reports which have determined the estrogenic modulation of TTCCs in breast cancer. Additionally, the expression of GPER is observed in normal breast tissues as well as breast tumors [188,189,190], however, the data regarding the involvement of GPER and LTCCs or TTCCs in estrogen signaling in cancer/breast cancer is also lacking.

#### 2.2.4. Endocrine and Reproductive Defects

Xenoestrogens and phytoestrogens by their action on VGCCs have the potential to cause endocrine and reproductive defects. Most of these effects were dependent on the type of environmental estrogen. The natural polyphenolic stilbenoid, resveratrol inhibited TTCC, and LTCC currents in the insulinoma cell line INS-1E, in a dose-dependent manner thereby contributing to the suppression of insulin release and thus displaying the potential to cause diabetes [191]. However, the diabetic potential is estrogen-dependent since another phytoestrogen quercetin actually protected against diabetes [192]. Quercetin also stimulated steroidogenesis in the MA-10 mouse Leydig tumor cell line via calcium influx through L-and TTCCs [193], which may be considered beneficial for male infertility but would be detrimental in children exposed to excess quercetin. Genistein, a phytoestrogen, inhibited sperm motility and acrosome reaction by inhibiting the TTCC current in the mouse spermatogenic cells of CD1 mice in a protein tyrosine kinase-independent manner. This study provided evidence of the potentially toxic effects of environmental estrogens on the male reproductive system [194]. Overall, it is observed that environmental estrogens have the potential to cause toxic or protective effects, but concentrations used in these acute exposure experiments may be well different from what exhibits toxic or protective effects and the data need to be carefully interpreted.

## 3. Conclusions and Perspectives

**Figure 1:**
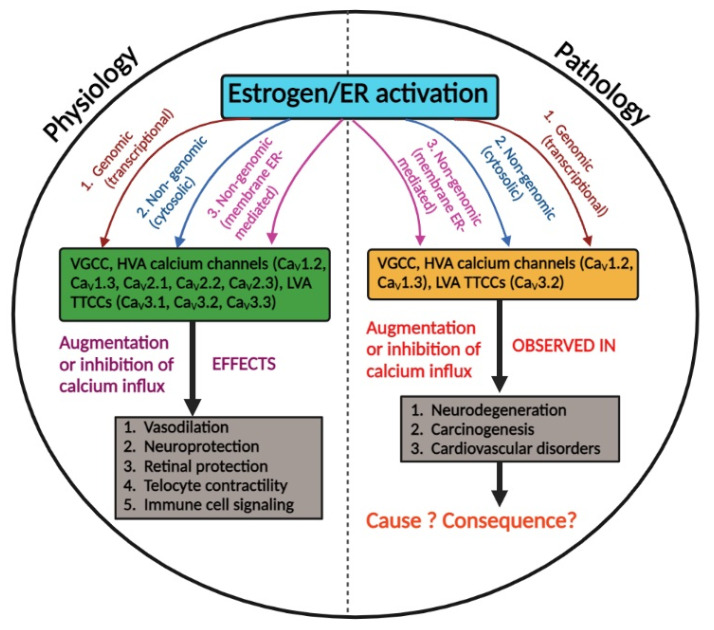
Overview of estrogenic modulation of VGCCs in physiology and pathology. Left: in physiological conditions, estrogen/ER activation modulates the calcium influx via VGCCs which contributes to various physiological functions. Right: estrogen/ER activation modulates the calcium influx via VGCCs which is observed in various disease conditions. However, whether this modulation causes the pathology or is merely a consequence requires further investigation.

ERs and VGCCs undoubtedly are key players in many physiological processes across varied tissues and are disease targets in breast cancer and neuronal/cardiac diseases, respectively. Overall, the data reviewed here suggest a very complex estrogen signaling involving modulation including both up- and downregulation of HVA and LVA calcium channels (Figure 1). It appears that, in most cases, inhibition of VGCCs was observed during acute application of high concentrations of estrogen though some exceptions exist. During acute application, most of the studies reported underlying non-genomic mechanisms that operate either via membrane receptors such as GPERs or cytoplasmic second messengers. However, very low concentrations of estrogen upon chronic exposure resulted in the potentiation of VGCCs mainly through genomic, ER-dependent mechanisms in vivo. There is still uncertainty regarding what governs the inhibition or upregulation of VGCCs by estrogen/ER. Perhaps more in vivo studies would be beneficial in understanding the physiological regulation of VGCCs by estrogen/ER and also shed light on the involvement of ER isoforms. So far, most of the regulation is described for ERα. We have also provided information on in vitro and in vivo systems that have been used to study such interactions and other systems/methods that can be potentially used to study such interactions (Table 4).

ER regulation of VGCCs also has consequences for pathologies where malfunction of both ERs and VGCCs is observed. Regulation of VGCCs by ERs suggests that perhaps targeting them both in cancer or other diseases such as neurodegenerative diseases may be beneficial but there is much to be explored in this regard. Estrogen inhibits VGCCs in neuronal systems to provide protection against calcium overload, but this regulation has not been exploited so far in pathological conditions. Recently shown co-occurrence of alterations in VGCCs and ERs in breast cancer patient tissues provides a prospective therapeutic opportunity where perhaps targeting them both would be beneficial. In diseases such as cancer, future therapies involving multiple targets would be beneficial to overcome resistance to therapies. However, as evidenced by a handful of studies, targeting VGCCs and ERs in an isoform-specific manner would be more effective since different isoforms can perform differential signaling in different subtypes of breast cancer. It was previously suggested that existing chemotherapeutic drugs provided better outcomes when used together with calcium channel blockers, the mechanism behind this was the cell cycle arrest of cancer cells by calcium channel blockers which could improve the effectiveness of the chemotherapeutic drugs. On a similar principle, calcium channel blockers together with ERBs may be investigated for their therapeutic benefits. Nevertheless, more studies are needed to understand the role of specific isoforms in pathological conditions be it cancer, neuronal or cardiovascular. To our knowledge, isoform-specific estrogenic modulation of TTCCs has not been investigated in breast cancer yet. Given that both show a causal relationship in breast cancer, it would be pertinent to determine their association with breast cancer. It would also be crucial to determine whether estrogen regulates TTCC activity in ER-dependent or an ER-independent manner. In addition, since few studies showed isoform-specific regulation of TTCCs by estrogen, it would be interesting to determine whether estrogen targets all isoforms or has a preferential selectivity for any particular isoform of TTCCs or VGCCs. In addition to therapeutics, co-expression of TTCC and ER isoforms can be useful biomarkers as demonstrated in breast cancer subtypes. The discovery of novel biomarkers is also essential for correct diagnosis, specific treatment, and prognosis.

Another area which lacks much information is the involvement of GPCRs and GPERs in the regulation of VGCCs by ERs. ER together with the human epidermal growth factor (HER2) receptor promotes signaling in breast cancer which is suggested to play an important role in the resistance to endocrine therapy [195]. Estrogen also stimulates insulin-like growth factor-1 (IGF-1) mRNA production in both normal breast tissues as well as breast cancer. This interaction of ER with HER2 and IGF-1 receptors is well documented [196,197,198]. However, at the moment, it is unclear whether this crosstalk of ER with other receptors can influence the regulation of VGCCs by ER. These interactions may have consequences for SERM therapy and may elucidate the mechanism of resistance in SERM therapy. It is also important in light of the fact that VGCCs are being suggested as a target in breast cancer [62] and other cancers as well where estrogen signaling is also very important.

We also made an interesting observation regarding the differential regulation of LTCCs by estrogen in the heart, based on the structural differences. In many cells such as neuronal cells and blood platelets, it is shown that estrogen signaling and ERs are linked to cellular microdomains such as lipid rafts and depleting lipid rafts can abolish the estrogen signaling. Since VGCCs and other ion channels also reside in lipid rafts, it would be interesting to observe if estrogen regulation of channels that are present in the lipid rafts is different from the channels located elsewhere on the membrane. This can be potentially achieved by super-resolution scanning patch clamp technique which allows us to image the cell surface microdomains such as T-tubules, neuronal cell bodies, caveolae, etc., [199] and then one can record ion channel currents from that microdomain in the presence and absence of estrogen.

Overall, the crosstalk between VGCCs and estrogen/ER is an important signaling pathway in many cell types, the full potential of which remains to be discovered and exploited.

## Figures and Tables

**Table 1:** Localization of voltage-gated calcium channel subtypes.

Channel	Current Type	Localization
Ca_V_1.1	L	Skeletal muscle [11]
Ca_V_1.2	L	Heart, smooth muscle, brain, pituitary, pancreatic β-cells, adrenal medulla [8,19,20]
Ca_V_1.3	L	Brain, pancreatic β-cells, medulla, kidney, ovary, cochlea [8,21,22]
Ca_V_1.4	L	Retina [10]
Ca_V_2.1	P/Q	Central nervous system, cerebellum, cochlea, adrenal medulla [16,23,24]
Ca_V_2.2	N	Brain, peripheral nervous system, adrenal medulla [23]
Ca_V_2.3	R	Central nervous system, pancreatic islets, adrenal medulla [18,23,24]
Ca_V_3.1	T	Brain, ovary, placenta, heart, peripheral nervous system, pancreatic β cells, adrenal medulla [25,26]
Ca_V_3.2	T	Heart, brain, kidney, pancreatic β cells, adrenal cortex [27,28]
Ca_V_3.3	T	Brain, pancreatic β cells [29,30]

**Table 2:** Estrogenic modulation of VGCCs in cardiovascular tissues in physiological conditions.

Effect of Estrogen on VGCCs	Experimental System	Mechanism of Action	Remarks
VGCC inhibition	Rabbit coronary [112] and basilar artery [113], vascular smooth muscle cells from Sprague Dawley rats [106], guinea pig ventricular myocytes [107]	Non-genomic/(not-specified)	Estrogen inhibited the calcium influx via VGCCs and thereby caused vasodilation. This inhibitory effect was reported to occur via a pertussis toxin-sensitive GTP-binding protein [113].
LTCC inhibition	Rat aortic smooth muscle (A7r5 cells) [111], mammalian smooth muscles [114], ventricular myocytes from rat, human and guinea pig [109,110], rabbit carotid artery [115], neonatal rat cardiac fibroblasts [108], HEK-293 cells transiently transfected with human Cav1.2α [121]	Non-genomic/receptor-independent	Estrogen reduced calcium influx through inhibition of LTCCs in various cardiovascular tissues.
	Right coronary artery from female Yorkshire pigs [126]	ER-dependent	Estrogen reduced the LTCC protein expression via ERα/ERβ dependent pathway. Estrogen binds to ERα/ERβ and alters the post-transcriptional regulation of LTCC.
TTCC inhibition	Neonatal cardiomyocytes from female Wistar rats [124]	Receptor-independent	TTCC downregulation by estrogen was mediated by ERK-1/2, 5 pathways.
LTCC and TTCC inhibition	A7r5 vascular smooth muscle cell line [120]	Not specified	Estrogen application attenuated the voltage-dependent calcium current (within 1–2 min) through TTCCs and LTCCs.
LTCC upregulation	Ventricular myocytes from adult male and female New Zealand white rabbits [127,128,129] and human iPSC derived cardiac myocytes.	ER-dependent	Physiological concentration of estrogen (1 nM) increased the calcium current only in cells from the base of the heart. This estrogenic effect could be correlated to humans [129].
	Rat ventricular myocytes, H9C2 cultured cells [130]	Membrane ER-dependent	Upregulation of calcium influx via LTCCs occurred via plasma membrane ER and by activation of PI3K, protein kinase B (Akt/PKB) and cAMP- response element binding protein (CREB) signaling.
	Mice left ventricular apical myocytes [131]	Non-genomic (GPR30)	Estrogen modulated the expression of genes related to the cAMP-PKA-LTCC pathway thereby contributing to sex differences in cardiac contraction. This acute estrogenic effect was concentration-dependent, sex-specific and mediated by GPR30.

**Table 3:** Estrogenic modulation of VGCCs in neuronal tissues in physiological conditions.

Effect of Estrogen on VGCCs	Experimental System	Mechanism of Action	Remarks
HVA VGCC inhibition	Female rat cortical neurons [145]	Non-genomic	Estrogen inhibited HVA calcium current in a rapid, reversible and concentration-dependent manner via PKC and PKA-dependent pathways.
N-and LTCC inhibition	Sensory neurons of female Sprague Dawley rats [141]	Non-genomic	First evidence of linking modulation of HVA L-and N-type calcium currents by estrogen to in vivo sensory modulation.
LTCC inhibition	Hippocampal zipper slices from female Fischer rats [142]	Not specified	Estrogen inhibited LTCC Ca_V_1.2, but not Ca_V_1.3.
	Neostriatal neurons from Sprague Dawley rats [140], hippocampal cells from Wistar rats [143], neuronal cells from Wistar rat cortex [146]	Non-genomic/membrane receptor mediated	Estrogen inhibited LTCCs via a non-genomic mechanism. ER antagonists or inhibitors of PKA/PKC did not affect the estrogen-mediated inhibition of HVA LTCCs, suggesting that more than one mechanism may be operating in neuronal tissues.
	GnRH producing GT-17 neuronal cells [153]	ER-dependent	Estrogen reduced the transcription of Ca_V_1.3 LTCC.
LTCC upregulation	Rat hippocampal neurons, hippocampal slices, and HEK-293 cells transfected with neuronal LTCCs [151]	ER-independent/membrane receptor mediated	Estrogen directly potentiated recombinant Ca_V_1.2 in the hippocampal neurons via an ER-independent mechanism through direct binding with a domain that overlaps the dihydropyridine-binding site [151].
L-and R-type VGCC upregulation	GnRH neurons from adult female mice expressing eGFP [152]	ERβ and GPR30	Estrogen rapidly increased the inward calcium currents through L-and R-type channels by activating ERβ and GPR30, respectively.
TTCC upregulation	Adult C57BL/6 mice hypothalamic arcuate nucleus [156], mice hypothalamic nuclei and pituitary [159]	ER-dependent	Estrogen-induced increase in mRNA expression of Ca_V_3.1 and Ca_V_3.2 in the hypothalamus was dependent on ERα and both (ERα and ERβ), respectively. However, in the pituitary, the estrogenic effect was dependent on the expression of ERα alone [159].
	Mice-GnRH neurons [158]	Membrane ER	All three TTCC isoforms are expressed in GnRH neurons and the estrogen-dependent upregulation of TTCC is membrane ER-mediated.
	Sprague Dawley rat ventromedial hypothalamic neurons [157], Guinea pig hypothalamus and pituitary neurons [155]	Not specified	Estrogen enhanced LVA calcium current in the absence of phenylephrine, an α_1_adrenergic agonist. In contrast, in its presence, augmentation of HVA calcium currents mediated by N-and L-type VGCCs was observed [157].
P/Q and TTCC upregulation	C57BL/6 mice pituitary [160]	ERα-dependent	Estrogen regulated P/Q and TTCCs via ERα-mediated pathway in the pituitary.

**Table 4:** Models/tools to study ligand-voltage-gated ion channel interaction.

In Vitro Models or Methods	
Experimental System/Tools	Potential Effects That Can Be Studied
Heterologous overexpression of ion channels in cell lines such as HEK293 and COS-7, primary cell lines.	Direct modulation of the voltage-gated ion channel by the ligand using patch-clamp technique (inhibition/augmentation). Alteration of mRNA and protein expression of ion channel upon treatment of cells with the ligand.
Native cell lines (e.g., INS-1E, PC12).	Direct modulation of the voltage-gated ion channel by the ligand in situ using patchclamp technique. Association of ligand-ion-channel interaction with cellular effects such as secretion, proliferation, contraction, etc.
**In Vivo/Ex Vivo Models or Methods**	
Transgenic ligand deficient models (e.g., ovariectomized (ER-deficient animal model), disease models (e.g., models for Parkinson’s or Alzheimer’s). Brain slices for patch clamp recording of intact (DRG) neurons. Whole organ systems such as Langendorff heart.	Electrophysiological recording of intact channel activity where interacting partners may be present such as in brain slices. Alteration of mRNA and protein expression of the endogenous channel upon treatment of tissues/animals with ligand coupled with physiological effects.
**Alternate Models or Methods**	
Economic zebrafish models	Imaging of ion channels in vivo (since embryos are transparent). Endogenous channel mRNA and protein expression in whole animals.
Super-resolution scanning patch-clamp	Microdomain-dependent estrogen regulation of ion channels.
Bio-layer interferometry (BLI)	Biomolecular interactions for investigating the binding sites of estrogen/ligand on ion channels in cell lysates.
Functional in-silico models (such as the recent A549 in-silico whole-cell ion current model)	The alterations in ion channels caused by various stimuli can be investigated with a digital model prior to experimental validation in the native background.

## Data Availability

All the data are published previously.
